# Per- and polyfluoroalkyl substances in waters associated with oil and gas development in the Denver Basin

**DOI:** 10.1038/s41598-025-33394-9

**Published:** 2026-02-07

**Authors:** Matthew S. Varonka, Aaron M. Jubb, Bonnie McDevitt, Jenna L. Shelton, Elliott P. Barnhart, Denise M. Akob, Isabelle M. Cozzarelli

**Affiliations:** 1 Geology, Energy & Minerals Science Center, U.S. Geological Survey, 12201 Sunrise Valley Dr. MS 954, Reston, VA 20192 USA; 2 Illinois State Water Survey, Prairie Research Institute, Champaign, IL 61821 USA; 3 Wyoming-Montana Water Science Center, U.S. Geological Survey, Helena, MT 59601 USA

**Keywords:** Energy, Hydraulic fracturing, Wastewater, Water quality, PFAS, Environmental monitoring, Geochemistry

## Abstract

Use of per- and polyfluoroalkyl substances (PFAS) in the petroleum industry could be a cause for concern due to the large volumes of produced water (PW) generated during oil and gas extraction, the reuse of these wastes in water-stressed regions, and adverse health outcomes related to PFAS exposures. However, PW PFAS characterization is nearly absent in the literature, and hydraulic fracturing (HF) chemical disclosures often omit the identities of additives as proprietary. Here we evaluate PFAS in PW samples from three petroleum wells in the Denver Basin during their first year of production. Total concentrations of targeted PFAS (Σ_40_PFAS) were < 35 ng/L in PW samples, with short-chain PFAS like perfluorobutanoic acid persisting throughout the sampled duration. Analysis of freshwater inputs for hydraulic fracturing (Σ_40_PFAS ~ 113 ng/L) and mixed fracture fluid (Σ_40_PFAS ~ 69 ng/L) indicated much of the targeted PFAS content was derived from the input water, and not from HF additives, however samples subjected to oxidation indicated the presence of PFAS precursors that would not be detected by targeted analysis. This study highlights that while PFAS content is low in the studied PWs, the potential for redistribution of PFAS in the environment may be a consideration for reuse applications.

**Supplementary Information:**

The online version contains supplementary material available at 10.1038/s41598-025-33394-9.

## Introduction

Per- and polyfluoroalkyl substances (PFAS) are persistent and widespread in the environment and are linked to a range of environmental and human health effects^1^. Sources of PFAS to the environment include both point sources (e.g., municipal and industrial effluents, landfill leachates) and distributed sources (e.g., household product and wastes, biosolid land application)^1–3^. Recently, there has been interest in PFAS occurrence in waters co-produced (produced water [PW]) during oil and gas (O&G) activities due to the large volumes of these waters generated annually (25.86 billion bbl)^4^ and efforts to reuse these waters beneficially (e.g., irrigation, supplementing streamflow, industrial use, aquifer recharge)^5–8^. PFAS can be introduced into O&G wells through contamination of input water used for mixing hydraulic fracturing (HF) fluids, in chemical formulations deliberately added to HF or workover fluids, or through leaching from flowlines and pipe joints, and can ultimately be incorporated into PW. Much of this PW is reinjected into disposal wells, but efforts to reuse PW for beneficial purposes necessitate PFAS characterization for sound risk management.

Previous studies focusing on chemicals added during HF^9^ have relied on the FracFocus Chemical Disclosure Registry (FracFocus), which includes industry self-reported data on fluid additives^10^. Few of the disclosures include PFAS chemicals, with approximately 1% of wells (674/60,986) reporting fluorochemical additives in the five-year period between 2019 and 2023^10^. One of the most common classes of fluorochemicals listed in the database is nonionic fluorosurfactants, which are used as emulsion stabilizers and wetting agents to increase O&G production,^11^ however these chemicals are commonly listed as proprietary with no associated Chemical Abstract Service Registry Number^10^. The mass and proportion of additives designated as proprietary in the database have been increasing in recent years^12,13^. These ambiguities have led to speculation that PFAS use in O&G operations is more common than reported,^10^ though this has been disputed by energy industry-backed groups^14^. Concerns over non-disclosure have also prompted a ban on the sale or distribution of O&G products with added PFAS in Colorado (HB22-1345) and the current consideration of regulations to mandate full disclosure chemical disclosure of fracture fluid additives in New Mexico.

Chemicals added during HF can return to the surface in PW, however, chemical characterization studies of PFAS in these wastes are extremely limited in the literature with only one study published to our knowledge. Jiang et al.^15^ analyzed one PW sample from a salt-water disposal well in the Permian Basin and detected five PFAS compounds with concentrations below the method reporting levels. Although data supporting PFAS in PW are limited, 37 PFAS compounds were identified in the surface waters, soils, and sediments of the Dagang Oilfield, China, suggesting broad PFAS contamination is possible in oil and gas fields^16,17^.

**Fig. 1:**
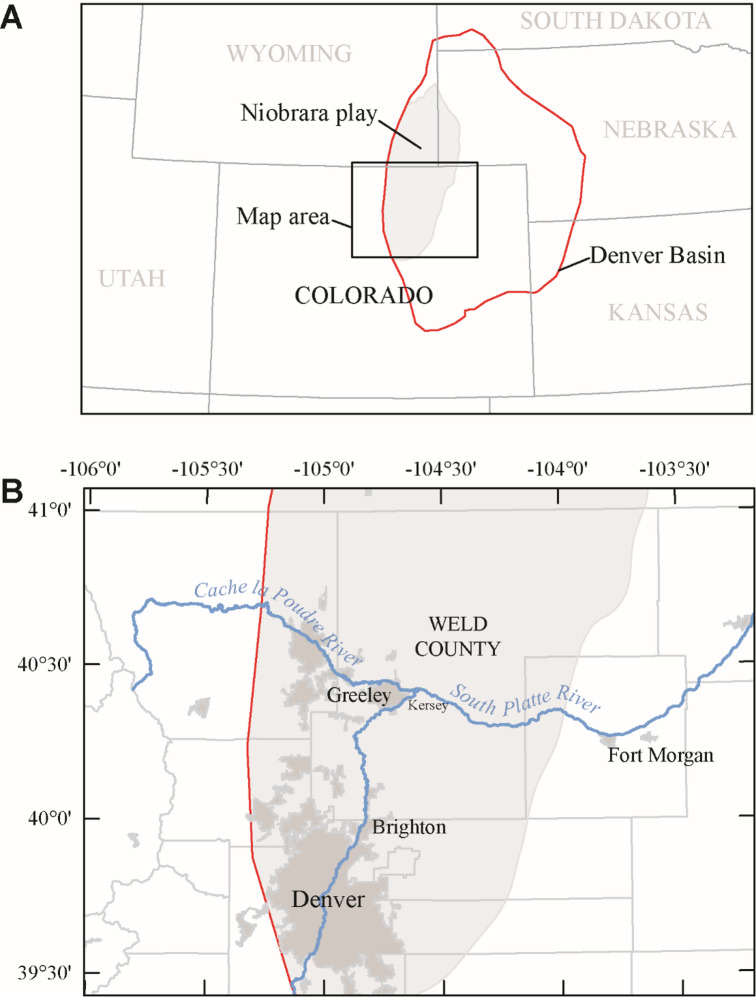
Maps of (**A**) the Niobrara play within the Denver Basin and (**B**) Weld County, Colorado and the South Platte River. Map generated with Esri ArcGIS Pro 3.4.3. Niobrara play boundary and Denver Basin boundary available from: U.S. Energy Information Administration. Maps: Oil and Natural Gas Exploration, Resources, and Production. https://www.eia.gov/maps/maps.php.

To address this data gap, here we evaluate PFAS in time-series PW samples (5 samples per well over the first year of production) from three active, unconventional petroleum wells (NWTS-1, NWTS-2, and NWTS-3) producing from the Niobrara Formation in the Denver Basin, northeastern Colorado (Fig. 1). As PFAS are not naturally occurring compounds and would need to be introduced into the system, concentrations, if detected, were expected to be greatest immediately after hydraulic fracturing in the flowback stage of production and trend toward zero as the composition of the PW shifted toward formation water. Disclosure reports for each well sampled include proprietary ingredients added during hydraulic fracturing, but do not specifically list any fluorochemicals. In addition to the time-series PW samples, samples of input water (freshwater stored in ponds prior to injection; Input Pond 1, Input Pond 2) and mixed hydraulic fracturing fluid (HF Fluid) were also analyzed. This study represents the first published effort to quantify PW PFAS content from flowback to formation water and investigate the possible sources of PFAS to O&G PW through analysis of hydraulic fracturing input waters and mixed fluids. Characterization of the PFAS content of these fluids can aid in assessing the risks of PW reuse.

## Results and discussion

**Fig. 2:**
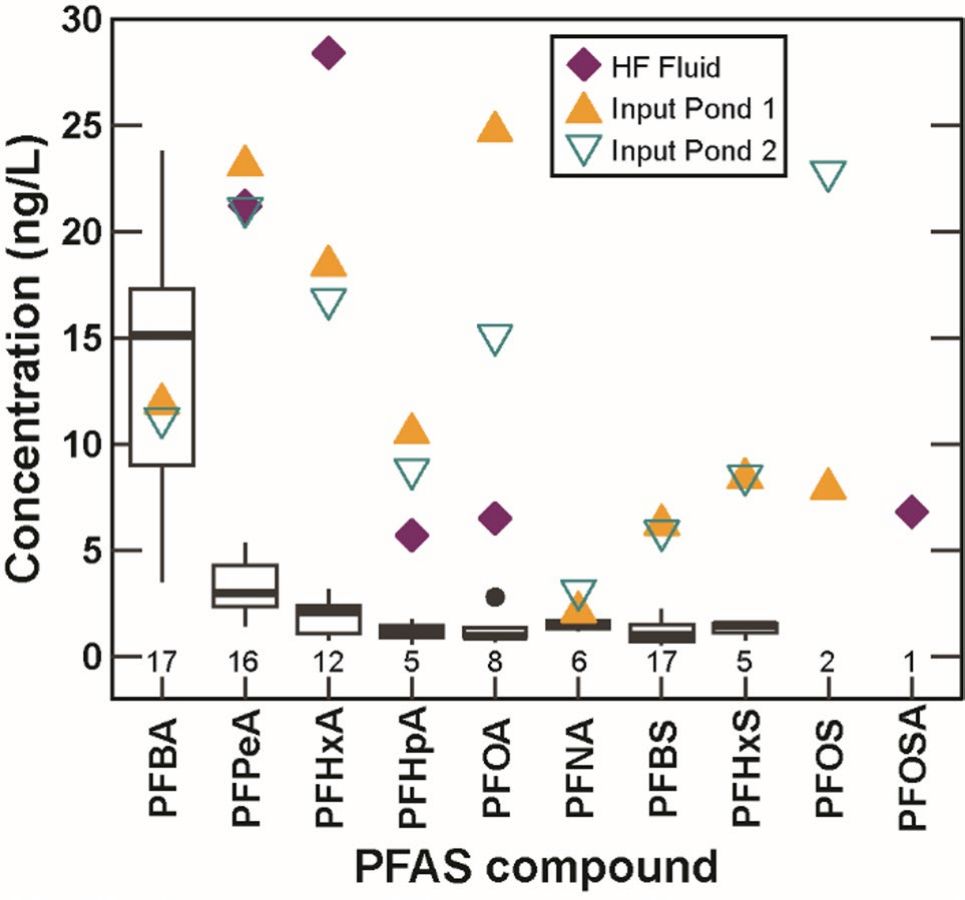
Concentration and detections of individual per- and polyfluoroalkyl substances (PFAS) in the time-series produced water samples (from wells NWTS-1, NWTS-2, and NWTS-3) (box plots), input ponds, and hydraulic fracturing (HF) fluid samples (shown as points). Numbers beneath box plots represent the total number of detections. Refer to Table S1 for a list of PFAS abbreviations.

Ten out of 40 targeted compounds were detected in the PW and HF input and mixed fluids, including perfluoroalkylcarboxylic acids (PFCAs), perfluoroalkyl sulfonic acids (PFSAs), and a perfluoroalkyl sulfonamide (Fig. 2, Table S2). Concentrations of individual compounds, estimated in cases where the concentration was less than the reporting limit, ranged from 0.543 to 28.4 ng/L. Perfluorobutanoic acid (PFBA) was detected in every time-series PW sample, ranging from 3.49 to 23.8 ng/L with a mean of 13.6 ± 5.9 ng/L. PFBA was also detected in both input ponds (~ 11 ng/L), however it wasn’t detected in the mixed hydraulic fracturing fluid, possibly due to higher detection limits for that particular sample due to analytical limitations (13 ng/L vs. 7.8 ng/L for the input ponds and < 3 ng/L for the time-series samples). Other compounds detected in the majority of time-series samples include perfluorobutanesulfonic acid (PFBS), perfluoropentanoic acid (PFPeA), and perfluorohexanoic acid (PFHxA), though these compounds, as well as other detected PFAS, were generally found at concentrations of less than 5 ng/L. Concentrations of PFCAs in the PW samples decreased with increasing molecular weight and PFSAs were found at lower concentrations than the PFCAs with the same alkyl backbone, possibly due to solubility differences. Total concentrations of targeted PFAS (Σ_40_PFAS) were ~ 113 ng/L in the input pond samples, ~ 69 ng/L in the mixed HF fluid, and a mean of 19.9 ng/L the time-series produced water samples (Fig. 2). Produced water Σ_40_PFAS concentrations (Table S3) were within an order of magnitude of PFAS measured in U.S. private and public drinking water supplies^18,19^ and surface waters,^20–22^ and ~ 2 orders of magnitude less than concentrations detected in wastewater treatment plant effluents^23^. In 2024, the U.S. Environmental Protection Agency (EPA) established National Primary Drinking Water Regulations maximum contaminant levels (MCL) for five individual PFAS (40 CFR Parts 141 and 142), four of which were detected in this study: perfluorooctanoic acid (PFOA, MCL 4 ng/L), perfluorooctanesulfonic acid (PFOS, MCL 4 ng/L), perfluorohexanesulfonic acid (PFHxS MCL 10 ng/L), and perfluorononaoic acid (PFNA, MCL 10 ng/L). Concentrations of these compounds in the time-series produced waters did not exceed the established MCLs, however PFOA did exceed the MCL in the hydraulic fracturing fluid and input waters, and PFOS concentrations exceeded the MCL in one of the input ponds samples. All concentrations were several orders of magnitude below the recently established acute freshwater aquatic life benchmarks established by the EPA (89 Fed. Reg. 81077 (October 7, 2024)).

With the exceptions of PFBA, PFHxA, and perfluorooctanesulfonamide (PFOSA), concentrations of individual PFAS compounds detected were highest in the input pond waters. Input water for HF is sourced from both groundwater and surface water in Weld County, Colorado,^10^ though the wells sampled in this study were fractured with only surface water and no produced water was introduced to the input water ponds for recycling. The South Platte River, which flows north through the metropolitan Denver area toward Greeley and then east to Fort Morgan, is the main source of surface water in Weld County and the predominant water source to these input ponds (Fig. 1). PFAS sources to surface waters include municipal wastewater treatment, industrial discharges, and urban and agricultural stormwater runoff, with research indicating land development as the primary driver of PFAS contamination^20,24^. Publicly available surface-water PFAS data are scarce for Weld County, but a 2020 survey measured a total PFAS concentration (for 18 analytes) of ~ 80 ng/L in the South Platte River near Kersey, the closest site to the sampled wells^25^. Contamination of the input water source prior to the mixing of HF fluid is likely one of the main contributors of PFAS to produced water in this study.

The mixed HF fluid had the highest concentration of PFHxA (28.4 ng/L) and the only detected level of PFOSA (6.8 ng/L) in all samples. The disclosure reports for the sampled wells include no specific fluorochemicals but do list between 12 and 14 proprietary ingredients per report including surfactants. However, given the relatively low concentrations of PFHxA and PFOSA, it is unlikely these compounds were deliberately added to the hydraulic fracturing mix, with contamination through contact with mixing equipment being a more likely contributor.

**Fig. 3:**
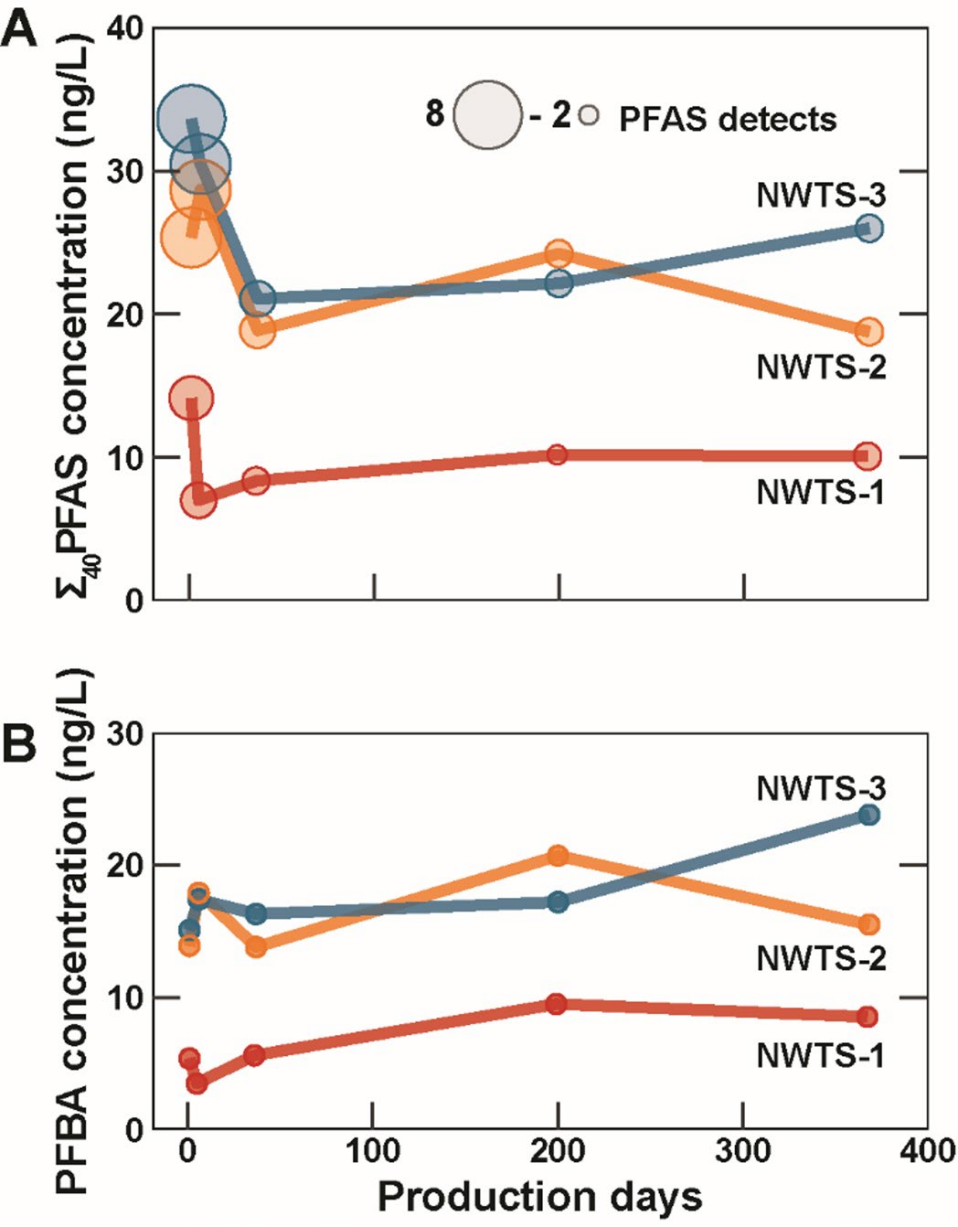
(**A**) Total concentration of targeted PFAS (Σ_40_PFAS) with number of detections and (**B**) perfluorobutanoic acid (PFBA) concentrations in the time-series produced water samples with the number of detections. Number of detections are indicated by circle size.

Concentrations of PFAS in the PW time-series samples were expected to be highest immediately after HF, when the influence of anthropogenic stimulation fluids derived from fresh surface water would dominate, and approach zero as more formation brine was produced. This transition from production of flowback of injected water to formation water is estimated to take approximately 200 days as indicated by the concentration of total dissolved solids (TDS) measured in the time series samples, with the most dramatic changes in TDS during the initial few weeks of production^26^. Indeed, both Σ_40_PFAS concentrations and the number of detections per sample were highest in the first week of production, however after the initial flowback period, Σ_40_PFAS concentration remained relatively constant throughout the first year of production (Fig. 3, Table S3). While concentrations of longer chain PFAS (PFHxA, PFOA, and PFNA) detected in the first week of production diminished with time, smaller chain PFAS (PFBA and PFBS) were detected in all 15 time-series produced water samples with relatively consistent concentrations over time (Fig. 3). Short-chain PFAS like PFBA could represent decomposition products of larger PFAS or PFAS precursor compounds within the system^27^. The detection of PFAS well beyond the initial flowback period points toward adsorption/desorption and/or degradation within the fracture network or leaching from a persistent secondary source. Previous studies have shown organic compounds like bisphenol F can leach from resin-coated hydraulic fracturing proppants into O&G produced waters over time,^28,29^ but no data on how PFAS can adsorb/desorb or directly leach from proppants is currently available in the literature. Additional sources of PFAS could include perfluorinated pipe sealants or greases in the produced water flow path, though further characterization of these materials would be needed to assess the potential of these materials as PFAS sources. Samples from wells NWTS-2 and NWTS-3, which have laterals approximately twice as long as well NWTS-1, had Σ_40_PFAS concentrations approximately twice that of NWTS-1, indicating well lateral length may be correlated with PW PFAS concentrations, however additional sampling would be needed to statistically confirm this possibility.

**Fig. 4:**
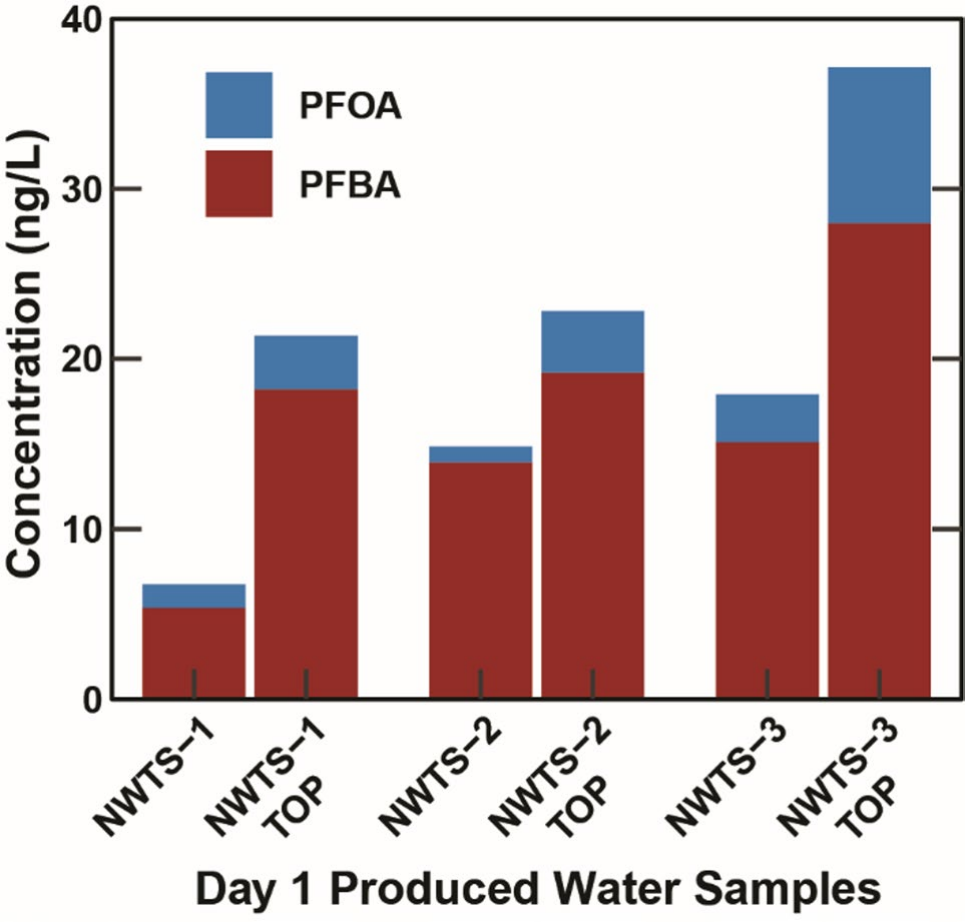
Perfluorooctanoic acid (PFOA) and perfluorobutanoic acid (PFBA) concentrations in day 1 produced water samples from the time-series wells for both the targeted PFAS method and the total oxidizable precursors (TOP) assay.

As the targeted PFAS analysis method only quantifies 40 PFAS compounds and thousands of PFAS are known to exist,^30^ an attempt to detect the presence of non-target PFAS within the produced water was conducted by subjecting samples from day 1 of production (flowback) to a total oxidizable precursor (TOP) assay which quantifies non-target perfluoroalkyl acids (PFAA) precursors. Due to detection limit differences and the need to exclude some data due to method blank detections, a direct comparison between the targeted Σ_40_PFAS concentrations and TOP concentrations is not possible. However, concentrations of PFBA and PFOA both increased with oxidation (Fig. 4). PFBA increased by 12.8, 5.3, and 12.9 ng/L (238, 38, and 86%) for NWTS-1, NWTS-2, and NWTS-3, respectively, with smaller magnitude increases in PFOA concentrations of 2.45, 2.67, and 6.36 ng/L (131, 448, and 228%). Substantial increases in the concentration of PFOA and PFBA in the oxidized samples and the PFOA + PFBA sum being greater than the targeted Σ_40_PFAS in NWTS-1 and NWTS-3 indicate the presence of PFAA precursors that were not detected by targeted analysis. As these precursors appear to be present in considerable concentrations, expanding the scope of analysis to include fluorotelomer alcohol (FTOH), non-targeted, and ultrashort-chain PFAS could be helpful for future studies.

**Fig. 5:**
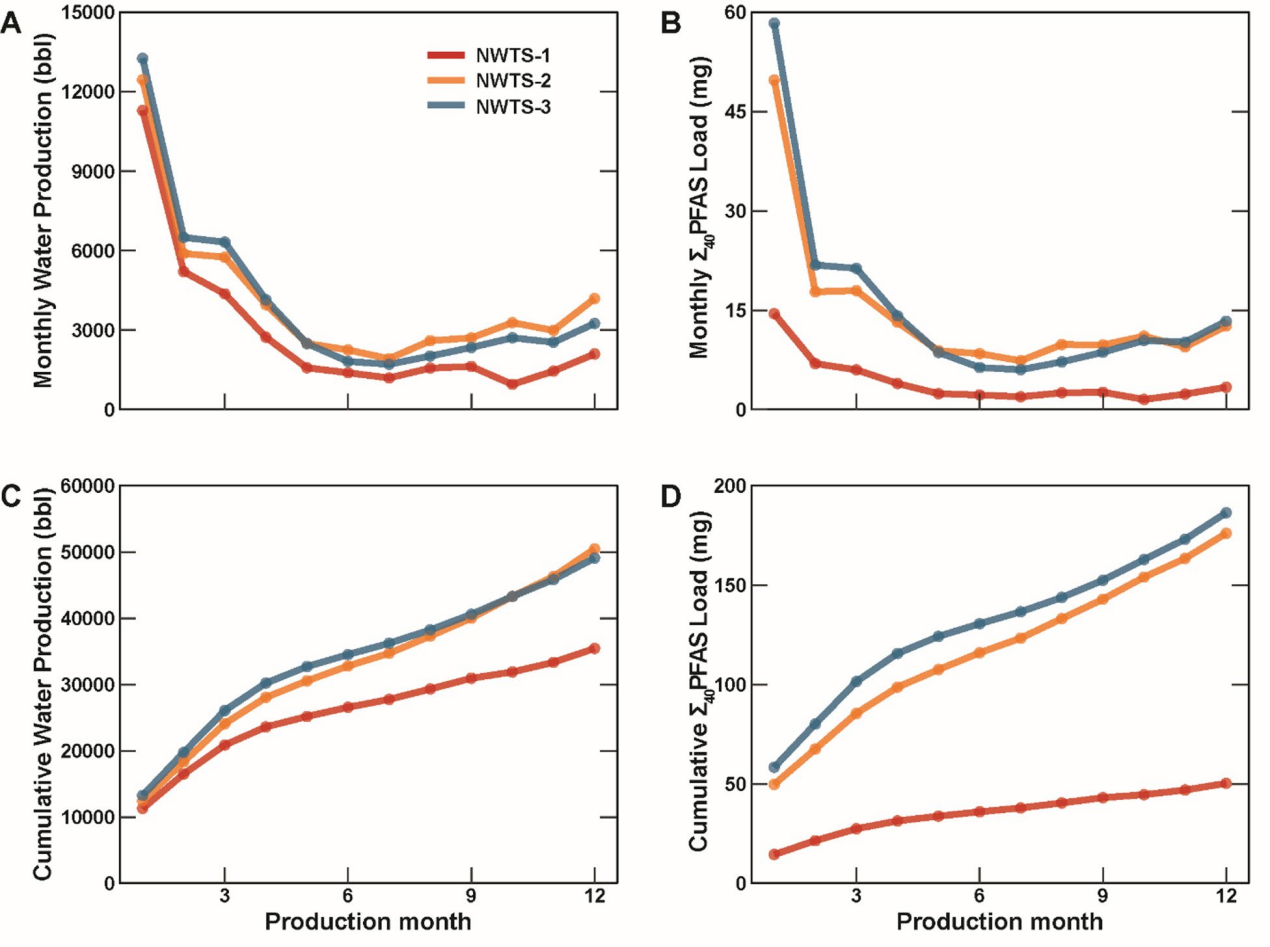
(**A**) Monthly water production from wells sampled with (**B**) estimated monthly Σ_40_PFAS loads and (**C**) cumulative water produced for the first year of production with (**D**) estimated cumulative Σ_40_PFAS loads. Data sourced from S&P Global Commodity Insights, 2023.

To gain perspective on the total loadings of PFAS from these wells in the first year of production, PW volumes were obtained from the S&P production database (Fig. 5A)^31^. Cumulative water production reached ~ 35,500, ~ 50,500, and ~ 49,000 bbl in the first year of production for NWTS-1, NWTS-2, and NWTS-3, respectively (Fig. 5C). Monthly mean Σ_40_PFAS concentrations for each well were estimated using LOESS models^32^ (calculated from targeted analyses), and these values were multiplied by the monthly water production to estimate monthly (Fig. 5B) and cumulative total PFAS loads (Fig. 5D). Cumulative Σ_40_PFAS loads over the first year of production were estimated at 50.3, 176, and 186 mg/y for NWTS-1, NWTS-2, and NWTS-3, respectively. These estimates are likely just a portion of the total PFAS load, however, as the estimates only account for the 40 targeted compounds in the EPA Draft 1633 method and significant concentrations of PFAS precursors were inferred from the TOP assay. While these PFAS load estimates are relatively low when compared to other wastewater streams, like wastewater treatments plant effluent which can contain hundreds of grams per day,^23^ the presence of PFAS could be an important consideration for the reuse of this voluminous wastewater stream.

This study represents the first effort in the literature to quantify PFAS in produced water during the transition from flowback to formation water and to assess the contribution of hydraulic fracturing input water and additives to PW PFAS concentrations. Major findings include: (1) relatively low concentration of PFAS were found in time-series PW samples despite the use of proprietary chemical additives during hydraulic fracturing, (2) contamination of hydraulic fracturing input ponds can be a significant source of PFAS to PW, (3) PFAS can persist in PW as production transitions from flowback to formation water, and (4) PFAS precursors are assumed present in these samples due to concentration increases after sample oxidation. This study does not represent the whole of O&G produced waters due to the limited number of wells sampled, and concentrations may represent minimum levels due to the extended time prior to analyses, subsampling protocol, and presence of precipitates. Further study could help to assess the potential risks of PW reuse from PFAS on a basin or national scale. Additional research on how PFAS interact with other constituents of produced water (e.g., salts, organics, metals, and naturally occurring radioactive materials) and the subsequent synergistic toxicity effects could also inform reuse risk.

### Methods

Time-series PW samples from three hydraulically fractured petroleum wells (NWTS-1, NWTS-2, and NWTS-3; *n* = 15, 5 samples per well from day 1 to 368 production days) and samples of the hydraulic fracturing input water (Input Pond 1 and Input Pond 2; *n* = 2, 1 sample per pond) and a mixed slickwater hydraulic fracturing fluid (HF Fluid; *n* = 1) were collected in 2018–2019 (total *n* = 18 samples). The wells were completed in the Niobrara Formation at a total vertical depth of approximately 6,300 ft (1900 m) and located in the Denver Basin, Weld County, Colorado; actual locations are anonymized per terms of the sampling agreement with the well operator (Fig. 1). Well NWTS-1 was hydraulically fractured using a slickwater fluid^33^ and had a lateral length of approximately 4,900 ft (1500 m), whereas wells NWTS-2 and NWTS-3 were hydraulically fractured using a crosslinked gel fracture fluid^33^ and had lateral lengths of approximately 9,800 ft (3000 m). Sampling methods and other geochemical parameters for these wells are described in detail by Jubb et al.^26^ Filtered, unacidified water samples were shipped on ice and stored (~ 3 °C) at the U.S. Geological Survey (USGS) in Reston, Virginia. Samples were subsampled in 2023 and 2024 and sent to SGS laboratories (SGS-Orlando, Orlando, Florida or SGS-AXYS, Sidney, British Columbia) for targeted PFAS analysis of 40 compounds (Table S1) via EPA Draft Method 1633^34^ (SGS-Orlando) and SGS AXYS MLA-110 methods (SGS-AXYS, equivalent to EPA Draft Method 1633). These results are available in a USGS data release^35^. A field blank collected simultaneously with the samples was also analyzed with no targeted PFAS detected. To evaluate the non-target PFAS in PW, three samples, one from each time-series well on day 1 of production, were subjected to a total oxidizable precursor (TOP) assay where unknown PFAS are oxidized to perfluoroalkyl acids (PFAA)^36^ using hydroxyl radical oxidation prior to analysis using SGS AXYS Method MLA-111. PFAS detections that were less than the reporting limit are considered estimated, and the values are included in this discussion. Compounds that were detected in both a sample and the associated method blanks have been omitted from this discussion but are included in the associated data release^35^. As the field samples were stored for approximately 4–5 years prior to analysis, were subsampled, and contained precipitates, the concentrations of PFAS reported likely represent minimum concentrations. While these limitations are not ideal, given the lack of produced water PFAS data, the persistence of these compounds in the environment, and the difficulty in obtaining O&G produced water and hydraulic fracturing input/mixed fluid samples, these results still represent a valuable contribution to the literature.

## Supplementary Information

Below is the link to the electronic supplementary material.

Supplementary Material 1

Supplementary Material 2

## Data Availability

The data used in this study are publicly available as a U.S. Geological Survey data release: Varonka, M.S. et al. Per- and polyfluoroalkyl substances (PFAS) in produced water from the Denver Basin. U.S. Geological Survey data release; https://doi.org/10.5066/P13SQVJA (2025).
