# Supplementary material for: Per- and polyfluoroalkyl substances in waters associated with oil and gas development in the Denver Basin
Source: Sci Rep. 2026 Feb 7;16:5743. doi: 10.1038/s41598-025-33394-9 (PMC12891480; doi:10.1038/s41598-025-33394-9)
Supplement: Supplementary file 1 — Supplementary Material 1 [file 41598_2025_33394_MOESM1_ESM.docx]

Supplementary information for

Per- and polyfluoroalkyl substances in waters associated with oil and gas development in the Denver Basin

Matthew S. Varonka^1^*, Aaron M. Jubb^1^, Bonnie McDevitt^1^, Jenna L. Shelton^2^, Elliott P. Barnhart^3^, Denise M. Akob^1^, and Isabelle M. Cozzarelli^1^

^1^Geology, Energy & Minerals Science Center, U.S. Geological Survey, Reston, VA, 20192, USA
^2^Illinois State Water Survey, Prairie Research Institute, Champaign, IL, 61821, USA
^3^Wyoming-Montana Water Science Center, U.S. Geological Survey, Helena, MT, 59601, USA

**This PDF file includes:**

Number of pages: 3

Number of tables: 3

**Table S1.** List of per- and polyfluoroalkyl substances and abbreviations included in Σ_40_PFAS.

PFBA Perfluorobutanoic acid

PFPeA Perfluoropentanoic acid

PFHxA Perfluorohexanoic acid

PFHpA Perfluoroheptanoic acid

PFOA Perfluorooctanoic acid

PFNA Perfluorononaoic acid

PFDA Perfluorodecanoic acid

PFUnA Perfluoroundecanoic acid

PFDoA Perfluorododecanoic acid

PFTrDA Perfluorotridecanoic acid

PFTeDA Perfluorotetradecanoic acid

PFBS Perfluorobutanesulfonic acid

PFPeS Perfluoropentanesulfonic acid

PFHxS Perfluorohexanesulfonic acid

PFHpS Perfluoroheptanesulfonic acid

PFOS Perfluorooctanesulfonic acid

PFNS Perfluorononanesulfonic acid

PFDS Perfluorodecanesulfonic acid

PFDoS Perfluorododecanesulfonic acid

4:2 FTS 4:2 Fluorotelomer sulfonate

6:2 FTS 6:2 Fluorotelomer sulfonate

8:2 FTS 8:2 Fluorotelomer sulfonate

PFOSA Perfluorooctanesulfonamide

N-MeFOSA 𝑁-Methylperfluorooctanesulfonamide

N-EtFOSA 𝑁-Ethylperfluorooctanesulfonamide

MeFOSAA 𝑁-Methylperfluorooctanesulfonamidoacetic acid

EtFOSAA 𝑁-Ethylperfluorooctanesulfonamidoacetic acid

N-MeFOSE 𝑁-Methylperfluorooctanesulfonamidoethanol

N-EtFOSE 𝑁-Ethylperfluorooctanesulfonamidoethanol

HFPO-DA Hexafluoropropylene oxide-dimer acid

ADONA 4,8-Dioxa-3H-perfluorononanoic acid

PFMPA Perfluoro-3-methoxypropanoic acid

PFMBA Perfluoro-4-methoxybutanoic acid

NFDHA Nonafluoro-3,6-dioxaheptanoic acid

9Cl-PF3ONS 9-Chlorohexadecafluoro-3-oxanone-1-sulfonic Acid

11Cl-PF3OUdS 11-Chloroeicosafluoro-3-oxaundecane-1-sulfonic acid

PFEESA Perfluoro(2-ethoxyethane)sulphonic acid

3:3 FTCA 3:3 Fluorotelomer carboxylate

5:3 FTCA 5:3 Fluorotelomer carboxylate

7:3 FTCA 7:3 Fluorotelomer carboxylate

**Table S2.** See Table S2.xlsx in the Supplementary information.

**Table S3.** Concentration of targeted PFAS (Σ_40_PFAS) with number of detections in the time-series produced water samples.

| Well ID | Production days | Σ_40_PFAS (ng/L) | Number of detections |
| --- | --- | --- | --- |
| NWTS-1 | 1 | 14.13 | 5 |
| NWTS-1 | 5 | 7.01 | 4 |
| NWTS-1 | 36 | 8.36 | 3 |
| NWTS-1 | 199 | 10.18 | 2 |
| NWTS-1 | 367 | 10.08 | 3 |
| NWTS-2 | 1 | 25.35 | 7 |
| NWTS-2 | 6 | 28.67 | 7 |
| NWTS-2 | 37 | 18.86 | 4 |
| NWTS-2 | 200 | 24.21 | 3 |
| NWTS-2 | 368 | 18.77 | 3 |
| NWTS-3 | 1 | 33.61 | 8 |
| NWTS-3 | 6 | 30.46 | 7 |
| NWTS-3 | 37 | 21.06 | 4 |
| NWTS-3 | 200 | 22.14 | 3 |
| NWTS-3 | 368 | 25.98 | 3 |
